# Global Routine Vaccination Coverage — 2017

**DOI:** 10.15585/mmwr.mm6745a2

**Published:** 2018-11-16

**Authors:** Kristin VanderEnde, Marta Gacic-Dobo, Mamadou S. Diallo, Laura M. Conklin, Aaron S. Wallace

**Affiliations:** ^1^Global Immunization Division, CDC; ^2^Department of Immunization, Vaccines and Biologicals, World Health Organization, Geneva, Switzerland; ^3^Division of Data, Research and Policy, United Nations Children’s Fund, New York.

Endorsed by the World Health Assembly in 2012, the *Global Vaccine Action Plan 2011–2020* (GVAP) (*1*) calls on all countries to reach ≥90% national coverage with all vaccines in the country’s national immunization schedule by 2020. This report updates previous reports (*2*,*3*) and presents global, regional, and national vaccination coverage estimates and trends as of 2017. It also describes the number of infants surviving to age 1 year (surviving infants) who did not receive the third dose of diphtheria and tetanus toxoids and pertussis–containing vaccine (DTP3), a key indicator of immunization program performance (*4*,*5*), with a focus on the countries with the highest number of children who did not receive DTP3 in 2017. Based on the World Health Organization (WHO) and United Nations Children’s Fund (UNICEF) estimates, global DTP3 coverage increased from 79% in 2007 to 84% in 2010, and has remained stable from 2010 to 2017 (84% to 85%). In 2017, among the 19.9 million children who did not receive DTP3 in the first year of life, 62% (12.4 million) lived in 10 countries. From 2007 to 2017, the number of children who had not received DTP3 decreased in five of these 10 countries and remained stable or increased in the other five. Similar to DTP3 coverage, global coverage with the first measles-containing vaccine dose (MCV1) increased from 80% in 2007 to 84% in 2010, and has remained stable from 2010 to 2017 (84% to 85%). Coverage with the third dose of polio vaccine (Pol3) has remained stable at 84%–85% since 2010. From 2007 to 2017, estimated global coverage with the second MCV dose (MCV2) increased from 33% to 67%, as did coverage with the completed series of rotavirus (2% to 28%), pneumococcal conjugate (PCV) (4% to 44%), rubella (26% to 52%), *Haemophilus influenzae* type b (Hib) (25% to 72%) and hepatitis B (HepB) (birth dose: 24% to 43%; 3-dose series: 63% to 84%) vaccines. Targeted, context-specific strategies are needed to reach and sustain high vaccination coverage, particularly in countries with the highest number of unvaccinated children.

In 1974, WHO established the Expanded Program on Immunization (EPI) to ensure that all children have access to four routinely recommended vaccines that protect against tuberculosis, diphtheria, tetanus, pertussis, polio, and measles (*4*): bacillus Calmette-Guérin vaccine (BCG), DTP, polio vaccine (Pol), and MCV. WHO and UNICEF derive national coverage estimates through an annual country-by-country review of all available data, including administrative and survey-based coverage (*5*,*6*); in general, only doses administered through routine immunization visits are counted.* DTP3 coverage by age 12 months is a key indicator of immunization program performance.

Despite increases in global DTP3 coverage from 79% in 2007 to 84% in 2010, DTP3 coverage has remained stable since 2010, estimated at 85% in 2017. In 2017, DTP3 coverage ranged from 72% in the WHO African Region to 97% in the Western Pacific Region (Table 1). National DTP3 coverage estimates ranged from 25% to 99%. Overall, 123 (63%) of 194 countries achieved ≥90% national DTP3 coverage in 2017, an increase from 117 countries (60%) in 2016. Among the 19.9 million children worldwide who did not receive 3 DTP doses during the first year of life, 12.4 million (62%) lived in 10 countries (Table 2). Among all children who did not complete the 3-dose DTP series in 2017, 13.7 million (69%) did not receive any DTP dose (“left out”) and 6.2 million (31%) started, but did not complete the DTP series (“dropped out”).

**TABLE 1:** Vaccination coverage, by vaccine and World Health Organization region — worldwide, 2017

Vaccine	No. (%) countries with vaccine in schedule	Coverage* (%)						
		Global	African	Americas	Eastern Mediterranean	European	South-East Asia	Western Pacific
BCG	158 (81)	88	80	92	86	92	91	97
HepB BD	105 (54)	43	10	69	34	41	44	85
HepB3	188 (97)	84	72	90	81	82	88	93
DTP3	194 (100)	85	72	91	81	94	88	97
Hib3	191 (98)	72	72	91	81	76	86	28
Pol3	194 (100)	85	71	90	81	93	88	97
Rota_last	96 (49)	28	46	68	30	24	9	1
PCV3	139 (72)	44	68	82	52	70	12	16
MCV1	194 (100)	85	70	92	81	95	87	97
RCV1	162 (84)	52	26	92	46	95	21	97
MCV2	167 (86)	67	25	74	67	90	77	94

**Abbreviations:** BCG = Bacille Calmette-Guérin vaccine; DTP3 = third dose of diphtheria and tetanus toxoids and pertussis-containing vaccine; HepB BD = birth dose of hepatitis B vaccine; HepB3 = third dose of hepatitis B vaccine; Hib3 = third dose of *Haemophilus influenzae* type b vaccine; MCV1 = first dose of measles-containing vaccine; MCV2 = second dose of MCV; PCV3 = third dose of pneumococcal conjugate vaccine; Pol3 = third dose of polio vaccine; RCV1 = first dose of rubella-containing vaccine; Rota_last = final dose of rotavirus vaccine series (number of doses to complete the series varies among vaccine products).

* BCG coverage based on 158 countries with BCG in the national schedule, whereas coverage for all other vaccines based on 194 countries (global) or all countries in the specified region. Administrative coverage is the number of vaccine doses administered to those in a specified target group divided by the estimated target population. During vaccination coverage surveys, a representative sample of households are visited and caregivers of children in a specified target group (e.g., aged 12–23 months) are interviewed. Dates of vaccination are transcribed from the child's home-based record, recorded based on caregiver recall, or transcribed from health facility records. Survey-based vaccination coverage is calculated as the proportion of persons in a target age group who received a vaccine dose.

**TABLE 2:** Number of surviving infants,* DTP3 coverage, and number of children not receiving DTP3 — worldwide and in countries with the highest number of children not receiving DTP3, 2007–2017

Area/Country	No. of surviving infants (millions)				DTP3 coverage (%)				No. not receiving DTP3 (millions)			
	2007	2012	2017	Change 2007 to 2017	2007	2012	2017	Change 2007 to 2017	2007	2012	2017	Change 2007 to 2017
Global	130.5	134.7	136.2	5.7	79	84	85	6	27.5	20.9	19.9	-7.6
Nigeria	5.6	6.3	6.9	1.3	42	42	42	0	3.3	3.6	4.0	0.7
India	25.9	24.6	24.3	-1.6	64	82	88	24	9.3	4.4	2.9	-6.4
Pakistan	4.5	4.9	5.1	0.6	54	64	75	21	2.1	1.8	1.3	-0.8
Indonesia	4.8	4.9	4.8	0.0	73	83	79	6	1.3	0.8	1.0	-0.3
Ethiopia	2.8	3.0	3.2	0.4	50	62	73	23	1.4	1.1	0.9	-0.5
DRC	2.4	2.8	3.2	0.8	70	75	81	11	0.7	0.7	0.6	-0.1
Angola	0.9	1.0	1.2	0.3	58	54	52	-6	0.4	0.5	0.6	0.2
Iraq	1.0	1.1	1.2	0.2	57	69	63	6	0.4	0.3	0.5	0.1
South Africa	1.1	1.1	1.1	0.0	82	65	66	-16	0.2	0.4	0.4	0.2
Afghanistan	1.0	1.1	1.1	0.1	63	67	65	2	0.4	0.3	0.4	0.0

**Abbreviations:** DRC = Democratic Republic of the Congo; DTP3 = third dose of diphtheria and tetanus toxoids and pertussis-containing vaccine.

* Number of children surviving to age 1 year.

Globally, the annual number of surviving infants increased by 4% (5.7 million) from 130.5 million in 2007 to 136.2 million in 2017.^†^ During this same period, global DTP3 coverage increased by 6% (from 79% to 85%), and the number of children who did not receive DTP3 decreased by 7.6 million (28%), from 27.5 to 19.9 million. Among the 10 countries with the highest number of children who had not received DTP3 in 2017, these trends varied. For example, during this period, the annual number of surviving infants decreased by 1.6 million (6%) in India, but remained stable or increased in nine of the other 10 countries (Table 2). DTP3 coverage increased in seven of these 10 countries and decreased in two. In Nigeria, the country with the largest number of children who had not received DTP3 in 2017, DTP3 coverage did not change during this period (Table 2). Among these countries, the number of children who had not received DTP3 decreased in the Democratic Republic of the Congo, Ethiopia, India, Indonesia and Pakistan, while in Afghanistan, Angola, Iraq, Nigeria, and South Africa, the number remained stable or increased.

In 2007, 9.3 million children in India and 3.3 million children in Nigeria did not complete the 3-dose DTP series (Figure). Although the population eligible for DTP3 declined by 6% in India and increased by 23% in Nigeria during 2007–2017, DTP3 coverage increased by 24% in India, but did not change in Nigeria. In 2014, Nigeria surpassed India as the country with the highest number of children who had not received DTP3 (3.72 million in Nigeria, 3.65 million in India).

**FIGURE:**
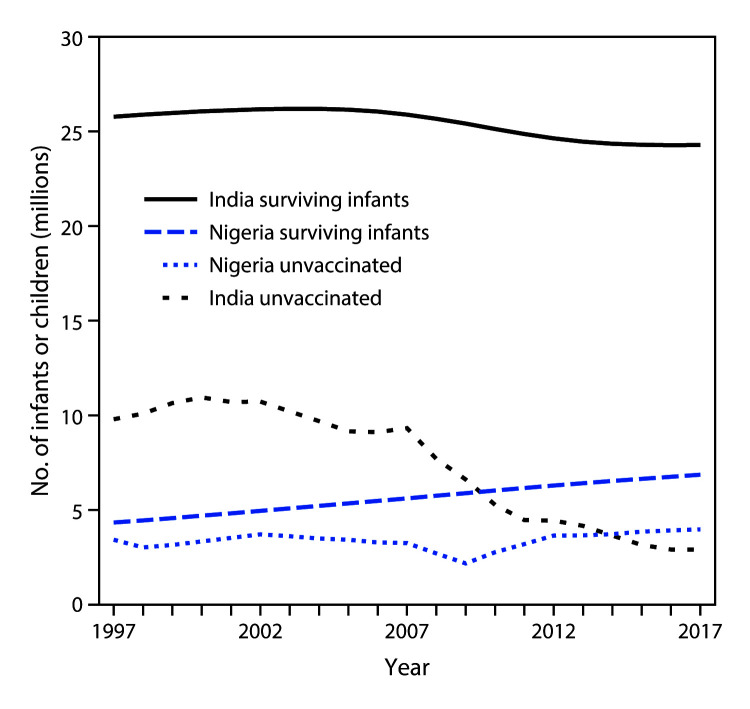
Number of surviving infants* and children who did not receive DTP3^†^ (unvaccinated) by age 1 year — India and Nigeria, 1997–2017 **Abbreviation:** DTP3 = third dose of diphtheria and tetanus toxoids and pertussis-containing vaccine. * Number of children surviving to age 1 year. ^†^ The number of children not receiving DTP3 is calculated based on yearly estimates of the number of surviving infants and DTP3 coverage rates.

Similar to DTP3, global MCV1 coverage increased from 80% in 2007 to 84% in 2010 and remained stable from 2010 to 2017 (85%). In 2017, MCV1 coverage ranged from 70% in the African Region to 97% in the Western Pacific Region (Table 1) and from 20% to 99% by country. Globally, 118 (61%) countries achieved the GVAP 2020 target of ≥90% national MCV1 coverage (*1*,*7*). Similar to DTP3 and MCV1, global Pol3 coverage increased from 81% in 2007 to 84% in 2010 and remained stable from 2010 to 2017 (85%). Global MCV2 coverage by the end of the second year of life increased from 16% in 2007 to 52% in 2017 and from 33% to 67% when older age groups (3–14 years) were included. MCV2 coverage by WHO region varied from 25% in the African region to 94% in the Western Pacific region, including in countries that have not yet introduced MCV2^§^ (Table 1).

Among new and underused vaccines, global coverage with the completed rotavirus series increased from 2% to 28% during 2007–2017. Coverage also increased for PCV^¶^ (4% to 44%), rubella (26% to 52%), Hib (25% to 72%), and HepB (birth dose: 24% to 43%; 3-dose series: 63% to 84%) vaccines (Table 1), as a result of both improvements in national coverages and new country introductions.

## Discussion

Substantial progress has been made in global vaccination coverage since the establishment of the EPI in 1974. Global coverage with DTP3 and MCV1 reached 85% in 2017, and global MCV2 coverage has doubled in the past decade. Challenges to achieving high routine immunization coverage remain, however, with only 63% and 61% of countries reaching the GVAP 2020 target of ≥90% national coverage for DTP3 and MCV1, respectively. Although global DTP3 coverage has remained stable for much of the past decade, this finding is not uniform across the 10 countries that are home to the highest number of children who have not received DTP3 in 2017, with increases in DTP3 coverage in seven countries, but decreased or unchanged coverage rates in three. Trends in MCV1 coverage from 2007 to 2017 are similar to those for DTP3, both globally and for the ten countries highlighted in this report (*7*).

Challenges to increasing and maintaining vaccination coverage need to be addressed in a country- and context-specific manner. In countries where coverage is increasing, continued investment in immunization programs will be critical for ensuring that gains are maintained. Historically, as DTP3 coverage has increased, the average annual rate of change in coverage has decreased, demonstrating the difficulty in maintaining positive annual growth, particularly at coverage levels above 90% (*8*). Specific strategies might be required to achieve coverage ≥90%. Among the 12.4 million children who have not received DTP3 living in the 10 countries with the most unvaccinated children, 73% (9.2 million) had not received any DTP doses, suggesting that many of the challenges lie with reaching the “left out,” those children who have not been reached by immunization programs. Rapid population growth might contribute to the challenges in maintaining or increasing coverage in countries where DTP3 coverage has declined or stagnated. Exploration of these population patterns and barriers to immunization at the subnational level might help to inform targeted interventions.

The findings in this report are subject to at least two limitations. Inaccuracies in vaccination coverage reporting at lower administrative levels and outdated national census data might result in over- or underestimation of administrative vaccination coverage. Second, parental recall errors could affect survey-based estimates of coverage (*5*,*7*).

Improvements in national immunization program performance are necessary to reach and sustain high vaccination coverage to increase protection from vaccine-preventable diseases for all children. Prioritizing countries with the highest number of unvaccinated children to implement targeted, context-specific strategies has the potential for a substantial impact on vaccination coverage globally.

**Summary**

**What is already known about this topic?**

Since 1974, global coverage with vaccines to prevent diphtheria, tetanus, pertussis, polio, and measles has increased from <5% to 85%.

**What is added by this report?**

Global coverage with the third dose of diphtheria and tetanus toxoids and pertussis-containing vaccine (DTP3), third dose of polio vaccine, and first dose of measles-containing vaccine has remained at 84%–85% since 2010. In 2017, 62% of children who did not receive DTP3 lived in 10 countries; positive trends in vaccination coverage (2007–2017) were observed in seven of these countries.

**What are the implications for public health practice?**

Prioritizing countries with the highest number of unvaccinated children to implement context-specific strategies has the potential to increase immunization coverage globally.

## Footnotes

* For a given vaccine, the administrative coverage is calculated by dividing the number of vaccine doses administered to those in a specified target group by the estimated target population. Doses administered through routine immunization visits are counted, but doses administered through supplementary immunization activities (mass immunization campaigns) generally are not. During vaccination coverage surveys, a representative sample of households are visited and caregivers of children in a specified target group (e.g., aged 12–23 months) are interviewed. Dates of vaccination are transcribed from the child's home-based record, recorded based on caregiver recall, or transcribed from health facility records. Survey-based vaccination coverage is calculated as the proportion of persons in a target age group who received a vaccine dose.

† Source for the number of surviving infants is the United Nations population projections database. https://population.un.org/wpp/.

§ Whereas BCG coverage is based on 158 countries with BCG in the national schedule, coverage for all other vaccines, including MCV2, is based on 194 countries for global estimates or on all countries in the specified region for regional estimates. In 2017, 86% of countries had MCV2 in their schedule.

¶ Global estimates for 3 doses of PCV available beginning in 2008; estimates shown are from 2008 to 2017.
